# Enriched rice husk biochar superior to commercial biochar in ameliorating ammonia loss from urea fertilizer and improving plant uptake

**DOI:** 10.1016/j.heliyon.2024.e32080

**Published:** 2024-05-29

**Authors:** Gunavathy Selvarajh, Huck Ywih Ch'ng, Norhafizah Md Zain, Lee Seong Wei, Jeng Young Liew, Siti Nuurul Huda Mohammad Azmin, Laila Naher, Palsan Sannasi Abdullah, Osumanu Haruna Ahmed, Mohamadu Boyie Jalloh, Issariyaporn Damrongrak

**Affiliations:** a Faculty of Agro Based Industry, University Malaysia Kelantan Jeli Campus, 17600, Jeli, Kelantan, Malaysia; b Department of Agriculture, Faculty of Applied Science, Lincoln University College, Selangor, Malaysia; c Universiti Islam Sultan Sharif Ali Sinaut Campus, Km 33, Jln Tutong Kampong Sinaut, Tutong, TB1741, Brunei Darussalam; d Faculty of Sustainable Agriculture, Universiti Malaysia Sabah, Locked Bag No. 3, 90509, Sandakan, Sabah, Malaysia; e Agricultural Program, Faculty of Science Technology and Agriculture, Yala Rajabhat University, Yala, 95000, Thailand

**Keywords:** Ammonia volatilization, Enriched biochar, Nutrient uptake, Urea fertilizer, Rice MR297 cultivar

## Abstract

Adding value to agricultural leftovers and turning them into biochar is a viable way to replenish soil nutrients and boost crop productivity. To further validate the efficacy of enriched rice husk biochar, an incubation study and a pot experiment were conducted: (1) to describe the effect of enriched rice husk biochar addition on soil total N, soil exchangeable NH_4_^+^ and available NO_3_^−^ and (2) to describe the effect of enriched rice husk biochar on improving N, P, K, Ca, and Mg uptake, use efficiency, and dry matter production of rice plants. The amount of NH_3_ loss that was considerably reduced by rice husk biochar at 5 and 10 t ha^−1^ was 34 % lower than the control. The availability of soil total N, exchangeable NH_4_^+^, available NO_3_^−^, available P, and exchangeable cations was greatly enhanced by the addition of rice husk biochar. Due to the effective nutrient uptake that occurs with an increase in soil nutrient level, the physical growth of the rice plant (height, tiller number, greenness, and panicle number) increeased significantly in treatments supplemented with 5 t ha^−1^ rice husk biochar. When rice plants were treated with 5 t ha^−1^ rice husk biochar, their absorption of N, P, and K increased by >80 %, respectively. The production of dry matter in rice plants increased as a result of the increased N intake. The application of 5 t ha^−1^ of rice husk biochar enhanced the soil nutrients by reducing NH_3_ loss and augmenting soil nutrients for efficient plant absorption, as demonstrated by the favourable enhancement of soil macro- and micronutrients and biomass of rice plants.

## 1 Introduction

One of the primary macronutrients that plants require for long-term growth is nitrogen (N). Nitrogen is often present in soil for plant uptake, but it is not sufficient since the decomposition of organic matter takes a longer time to release N. Therefore, in order to meet the requirement for plant nutrients in accordance with their growth stages, external inputs like N fertilizer must be administered. Urea fertilizer is a common type of N fertilizer used in rice fields because it is inexpensive and easily obtainable. However, application of urea fertilizer by the surface broadcasting method leads to urea volatilization in the form of ammonia (NH_3_) [1,2]. This is the challenge of surface applied urea, where, immediately upon contact with water, urease in the soil quickly converts the urea to NH_3_ [3]. The emission of NH_3_ gas will increase the environmental temperature and contribute to global warming. Apart from increasing environmental temperature, the volatilization of urea has a great impact on rice plants. The amount of N that rice plants can absorb are mostly reduced *via* NH_3_ volatilization. The irregular growth of rice plants caused by insufficient N has a direct impact on the yield and quality of the rice plants. Additional urea fertilizer needs to be used in order to make up for the N loss. This approach is costly, and over application can cause soil acidification [4] and water pollution. Therefore, it's critical to reduce the amount of NH_3_ lost as a result of the applied urea by using a long-term, sustainable strategy, such as adding organic amendments to rice fields.

Organic amendments such as biochar can be integrated with urea fertilizer to mitigate the problem of environmental pollution and fertilizer consumption by minimizing NH_3_ volatilization. Agricultural wastes, such as rice husk, which are widely available, can be used to produce biochar. As equal as the production of rice, the by-product of rice residues produced from the de-husking process is also increasing in milling factories. The Malaysian Ministry of Agriculture estimates that the country produces about 408,000 metric tonnes of rice husk every year [5]. The rice husk is considered to have no economic value. Besides, it was accumulated, and there is a lack of proper management to dispose the waste. The waste is usually burned or dumped in landfills. During the burning process, the carbon dioxide gas released into the environment has an adverse effect on the environment itself and human health [6]. Hence, a sustainable approach is needed to manage the waste without affecting the quality of the environment. Hence, the rice husk can be turned into a useful biochar product.

Carbon-rich solid biochar is obtained by pyrolyzing plant material at temperatures between 300 and 700 °C while providing a restricted amount of oxygen. Biochar presents itself as a novel, environmentally benign, and reasonably priced carbon material with a wide range of potential uses. Qian et al. [7] stated that biochar incorporation in soil can improve soil quality and crop production. It has been applied as a soil amendment to improve the physical characteristics of the soil, increase its capacity to store water, and enable it to absorb nutrients from the soil [8,9]. Lone et al. [10] stated that biochar influences soil N cycles by increasing the retention of inorganic N. Due to its vast surface area and multitude of pores, biochar can hold onto inorganic N such as NO_3_^−^ and NH_4_^+^ for extended periods of time [11]. The surface area and porosity of biochar are the primary factors that influence its adsorption rate. Retaining inorganic nitrogen is essential for effective plant absorption. Additionally, the increased CEC of biochar accelerates the rate at which nutrients adsorb into soil [12]. Because of these characteristics, biochar has a great deal of potential to reduce NH_3_ volatilization and improve soil nutrient retention, particularly N.

However, little is known about how incorporating rice husk biochar into the soil will lower NH3 volatilization in farming areas. To manage urea fertilizer application in the rice field sustainably, a detailed analysis is required to extrapolate the findings. This interaction occurs in the presence of soil, rice husk biochar, and rice plants. Therefore, the goals of this work were to: (i) apply enriched rice husk biochar to enhance soil total N, soil exchangeable NH_4_^+^, and soil available NO_3_^−^; and (ii) improve rice plant dry matter production and N, P, K, Ca, and Mg uptake and use efficiency.

## 2 Materials and methods

### 2.1 Soil sampling and selected chemical characterization

Before the experiment began, soil samples were taken to conduct an initial assessment of the soil. After being gathered, the soil samples were crushed, air dried, and sieved through a 2 mm sieve. A digital pH meter was used to measure the pH of the soil at a soil:water ratio of 1:10 [13]. Using the loss-on-ignition approach, total C, ash content, and soil organic matter were estimated [14]. The total amount of N was determined using the Kjeldahl method [15]. After the exchangeable cations (Ca, Mg, K, and Na) in the soil were determined, the cations were determined using atomic absorption spectroscopy (AAS) (Analyst 800, PerkinElmer, Norwalk, USA). Mehlich no. 1 double acid method was employed to extract the available P [16], which was then determined using the molybdenum blue method [17]. A UV-VIS spectrophotometer (Thermo Scientific Genesys 20, USA) operating at 882 nm wavelengths was used to analyse the produced blue colour. Soil CEC was computed using the ammonium acetate leaching method [18]. The acid-base titration method suggested by Rowell [19] was used to calculate the exchangeable Al^3+^ and acidity. Using the method described by Keeney and Nelson [20], exchangeable NH_4_^+^, and available NO_3_^−^ were extracted, and the quantities of the ions were then estimated by steam distillation [14].

### 2.2 Rice husk sampling and selected chemical measurements

The rice husks were obtained from Pasir Puteh Rice Mill in Kelantan, Malaysia. The recovered rice husk was subjected to pH and total N measurements [13,15]. The single dry ashing process was used to extract the Ca, Mg, Na, P, and K from rice husk [14]. The blue colour that emerged following the molybdenum blue method was evaluated using a UV-VIS Spectrophotometer (Thermo Scientific Genesys 20, USA) in order to determine the overall P content [17]. The concentrations of Ca, Mg, Na, and K were estimated using an Analyst 800 (PerkinElmer, Norwalk, USA); the total P content was estimated using the molybdenum blue method. The soil's organic matter, ash content, exchangeable NH_4_^+^, available NO_3_^−^, and CEC were analysed using the previously indicated analysis techniques.

### 2.3 Production, characterisation, and enrichment of rice husk biochar

For the manufacturing of biochar, a 110 L airtight drum and a 200 L cylindrical kiln with detachable chimney tops were built. Rice husk was added in the 110 L kiln followed by a tight close with the screw cap, and was placed at the centre of the 200 L kiln. A fire was started at the base of the 200 L kiln, and it burned for 4 h at a temperature of 300–400 °C. The pile of rice husk biochar was collected after the kilns allowed to cool for additional 2 h. The biochar was enriched by soaking it for seven days in a 5 % solution of chicken manure slurry, a by-product of the chicken industry. After drying, the biochar was stored in a large container for later use. In order to increase the biochar's pore size, change its surface area, and increase its nutrient content, the enrichment process using chicken manure slurry was essential [21,22]. The biochar was characterized using techniques akin to those described in Section 2.1 following the enrichment process. The morphology, surface area, pore volumes, and pore sizes of the enriched rice husk biochar were examined through microanalysis using BET (Quantachrome ASIQ060111-6, USA) and scanning electron microscopy attached to energy dispersive X-ray spectroscopy analysis (SEM-EDX JEOL JSM-6400).

### 2.4 Incubation study for NH_3_ measurement

A 250 mL conical flask was filled with soil and various rates of enriched rice husk biochar (5, 10, 15, and 20 t ha^−1^) before 175 kg ha^−1^ of urea was added. To test its effectiveness in minimizing NH_3_ loss, the produced enriched biochar was compared with a commercial biochar potting media. In one treatment, 100 % commercial biochar potting media was applied, while in another treatment, 50 % soil and 50 % commercial biochar potting media were mixed thoroughly. The commercial biochar potting media was then supplemented with urea fertilizer. To create a waterlogged environment, more water was added. Throughout the incubation study period, the water level in the conical flask was marked and kept 3 cm above the soil. In order to calculate the amount of NH_3_ loss from the applied urea, the boric acid solution was changed every 24 h and back-titrated with 0.01 M HCl. The measurement was carried out until 1 % of the added N in the system's NH_3_ was reached [23]. The pH, exchangeable NH_4_^+^, and available NO_3_^−^ of the soil samples were measured after the NH_3_ volatilization study period. Table 1 lists the evaluated treatments, which were set up in a completely randomized design (CRD) with three replications.

**Table 1:** Treatments evaluated in ammonia volatilization and pot study.

Treatment	Treatments evaluated in ammonia volatilization study	Treatments evaluated in pot study
CK0	100 g soil only (Negative control)	5 kg soil (Negative control)
CK1	100 g soil + 175 kg ha^−1^ urea (Positive control)	5 kg soil + 175 kg ha^−1^ urea +97.8 kg ha^−1^ CIRP + 130 kg ha^−1^ MOP (Positive control)
RHB1	100 g soil + 175 kg ha^−1^ urea +5 t ha^−1^ enriched rice husk biochar	5 kg soil + 175 kg ha^−1^ urea +97.8 kg ha^−1^ CIRP + 130 kg ha^−1^ MOP + 5 t ha^−1^ enriched rice husk biochar
RHB2	100 g soil + 175 kg ha^−1^ urea +10 t ha^−1^ enriched rice husk biochar	5 kg soil + 175 kg ha^−1^ urea +97.8 kg ha^−1^ CIRP + 130 kg ha^−1^ MOP + 10 t ha^−1^ enriched rice husk biochar
RHB3	100 g soil + 175 kg ha^−1^ urea +15 t ha^−1^ enriched rice husk biochar	2.5 kg soil + 2.5 kg commercial biochar potting media +175 kg ha^−1^ urea +97.8 kg ha^−1^ CIRP + 130 kg ha^−1^ MOP (50 % soil + 50 % commercial biochar potting media)
RHB4	100 g soil + 175 kg ha^−1^ urea +20 t ha^−1^ enriched rice husk biochar	5 kg commercial biochar potting media+ 175 kg ha^−1^ urea +97.8 kg ha^−1^ CIRP + 130 kg ha^−1^ MOP (100 % commercial biochar potting media)
CB2	50 g soil + 50 g commercial biochar potting media +175 kg ha^−1^ urea	Excluded in pot experiment
CB1	100 g of commercial biochar potting media +175 kg ha^−1^ urea	Excluded in pot experiment

For the NH_3_ loss incubation experiment, a close-dynamic air flow system was employed [[24], [25], [26]]. Two 250 mL conical flasks are part of the exchange chamber in the system; one contains a soil mixture and the other contains 75 mL of boric acid. Each flask had an inlet and an output pipe installed and was stoppered. The inlet of the chamber was fitted with an air pump and a water supply. By means of pipe tubing, the outflow was connected to the boric acid solution trap. The purpose of this arrangement is to provide air to the soil and stop NH_3_ from evaporating away.

### 2.5 Pot experiment

A pot experiment was conducted in a netted house on the Universiti Malaysia Kelantan Jeli Campus in Malaysia following the completion of the laboratory NH_3_ loss incubation experiment. Just five treatments from the NH_3_ loss incubation trial were selected to be further evaluated in the pot experiment based on their most promising results (Table 1). Treatments utilizing enriched rice husk biochar with 15 and 20 t ha^−1^ were not included in the pot experiment. The findings of the NH_3_ volatilization incubation study shown that, in contrast to the application of rice husk biochar at 5 and 10 t ha^−1^, the use of 15 and 20 t ha^−1^ did not significantly reduce NH_3_ loss (Table 3). Therefore, low application rates of rice husk biochar (5 t ha-1 and 10 t ha-1) were used. In order to assess the effectiveness of rice husk biochar in mitigating nitrogen loss, preserving soil nutrients, and improving plant nutrient uptake, treatments with soil only, soil + urea, 50 %, and 100 % commercial potting medium were carried forward to a pot experiment.

**Table 2:** Selected soil, rice husk, and enriched rice husk biochar physico-chemical properties.

Property	Soil	Rice husk	Enriched rice husk biochar
pH	5.5	6.5	9.1
EC (dS m^−1^)	0.022	NA	NA
Texture	Sandy Clay Loam	NA	NA
Soil organic matter (%)	6.24	NA	NA
Total C (%)	3.62	NA	NA
Ash content (%)	6.4	48.4	34.4
Cation exchange capacity (cmol_c_ kg^−1^)	5.4	34.5	66.6
Ammonium (ppm)	89	NA	NA
Nitrate (ppm)	30	NA	NA
Total N (%)	0.07	0.25	0.33
Available P (mg kg^−1^)	0.385	9.8	14.3
Available K (cmol_c_ kg^−1^)	0.084	1945	4925
Available Ca (cmol_c_ kg^−1^)	0.10	320	1048
Available Mg (cmol_c_ kg^−1^)	0.082	2186	508
Available Na (cmol_c_ kg^−1^)	0.024	59.3	256
Available Fe (cmol_c_ kg^−1^)	0.091	NA	NA
Exchangeable acidity (cmol_c_ kg^−1^)	0.7	NA	NA
Exchangeable Al (cmol_c_ kg^−1^)	1.14	NA	NA

**Note:** NA indicates not available.

**Table 3:** Effect of treatments during ammonia volatilization study on soil pH, exchangeable NH_4_^+^, exchangeable NO_3_^−^ and total NH_3_ loss.

Treatments	pH (water)	NH_4_^+^ (ppm)	NO_3_^−^(ppm)	Total ammonia loss (%)
**CK0**	5.53 ± 0.06^a^	106.67 ± 12.01^a^	32.67 ± 1.52^a^	0.00 ± 0.00^a^
**CK1**	6.23 ± 0.12^b^	256.67 ± 29.63^b^	37.67 ± 1.53^a^	44.52 ± 2.05^d^
**RHB1**	8.06 ± 0.06^e^	446.47 ± 3.33^e^	56.30 ± 0.88^b^	29.18 ± 0.07^b^
**RHB2**	7.95 ± 0.02^de^	464.33 ± 26.31^e^	56.00 ± 2.03^b^	29.44 ± 0.16^b^
**RHB3**	7.88 ± 0.02^de^	383.33 ± 31.80^de^	46.33 ± 0.88^b^	33.92 ± 1.05^c^
**RHB4**	7.75 ± 0.03^d^	335.00 ± 27.84^cd^	41.0 ± 0.58^b^	32.84 ± 0.77^bc^
**CB2**	7.38 ± 0.03^c^	270.00 ± 5.77^b^	30.33 ± 3.75^a^	44.06 ± 0.09^d^
**CB1**	7.36 ± 0.07^c^	221.00 ± 23.26^b^	30.34 ± 2.33^a^	48.83 ± 0.21^e^

Mean values within column with different letter(s) indicate significant difference between treatments by Tukey's test at p ≤ 0.05. Columns represent the mean values ± SE.

Rice plants (cultivar MR297) was served as the test crop in the pot experiment. The seedlings were placed in pots measuring 23 cm in height, breadth, and diameter. The pots were then filled with 5 kg of soil that had been sieved with a mesh size of 5 mm. Before being planted, MR297 rice seeds were allowed to sprout on a plastic tray that was filled with germination medium. Before the 7th day, rice seedlings were transplanted into the pot, the soil was well mixed with the enriched rice husk biochar at a rate of 5 t ha^−1^ and 10 t ha^−1^ 24 h earlier. Three rice seedlings per pot correspond to three seedlings per hill [27]. Each pot's water level was kept at 3 cm above the soil's surface. Urea (46 % N), Christmas Island Rock phosphate (32 % P_2_O_5_), and muriate of potash (60 % K_2_O) were used as N, P, and K fertilizers. The fertilizers are applied at rates of 175 kg ha^−1^, 97.8 kg ha^−1^, and 130 kg ha^−1^, respectively, after the 7th day rice seedlings have been transplanted. These rates were in accordance with the standard fertilizer application of the Muda Agricultural Development Authority, Malaysia [28] with the exception that the amount of urea used for each pot of 5 kg of soil was increased from 151 to 175 kg ha^−1^. At 7, 30, and 55 days after transplantation (DAT), the fertilizer was surface-applied in three equal portions. Table 1 provides a list of the treatments assessed in the pot trial.

In a net house, the pot experiment was conducted using a completely randomized design with three replications. Up until the heading stage (70 days), the plants were constantly inspected and monitored. The plants were harvested at 70 DAT. This is because the amount of soil utilized in the pots was insufficient to support the rice plants through the flowering and ripening stages, making it economically impractical to estimate the rice yield based on pot trials [29].

At the heading stage (70 DAT), the plant's height was measured using a measuring tape. The plants' degree of greenness was measured using the SPAD Meter 502-nm. The percentage of the rice plant greenness values over the control treatment was computed. The counts of panicles and tillers were made, and the findings were noted. The aboveground plant components were gathered and dried in an oven preheated to 60 °C to ensure a consistent weight [30]. The total contents of N, P, K, Ca, and Mg were estimated after the oven-dried plant samples were ground using a grinding machine. The total P and K were extracted from the plant tissues using the single dry ashing method, whereas the total N was determined using the Kjeldahl method. The molybdenum blue colorimetric method was used to estimate total P in the filtrates, whereas the AAS method was used to estimate total K, Ca, and Mg. The concentrations of N, P, K, Ca, and Mg in leaves were multiplied by the dry weight of the rice plants to determine the quantity of N, P, K, Ca, and Mg absorbed by the plants. Using Dobermann's approach [31], the efficiency of the nutrients used by the rice plant was estimated as follows:

Nutrientuptake=Concentrationofnutrient(%)×Oven−driedweightofsample(g)

$$\text{Nutrient}\;\text{use}\;\text{efficiency}=\frac{A-B}{R}\times 100$$

where,

A = nutrient uptake by plant from fertilized soil.

B = nutrient uptake by plant from unfertilized soil.

R = applied fertilizers rate.

As soon as the plants were harvested at 70 DAT, soil samples were sampled from the pots. The soil samples were crushed, allowed to air dry, and then sieved using a 2 mm sieve. The soil samples were tested for pH, EC, total N, accessible P, total organic matter, total C, exchangeable acidity and Al, exchangeable cations (K, Ca, Mg, Zn, and Fe), and total organic matter using the procedures outlined in section 2.1.

### 2.6 Statistical analyses

All the data were statistically analysed using SPSS software, version 24.0 (SPSS Inc., US). One-way analysis of variance (ANOVA) was used to determine how varying rates of enriched rice husk biochar addition affected the results. Tukey's HSD test was used to separate significant differences between treatments, and a difference was deemed significant at p<0.05.

## 3 Results

### 3.1 Characteristics of soil and rice husk biochar

Table 2 displays the selected physical and chemical characteristics of the soil. The pHofthesoilwas5.5, making it acidic. Exchangeable acidity, Al, and Fe were found to be higher in the soil in accordance with soil acidity. It was discovered that the soil's total available N, P, NH_4_^+^, NO_3_^−^, K, Ca, Mg, and Na were generally low.

**Fig. 1:**
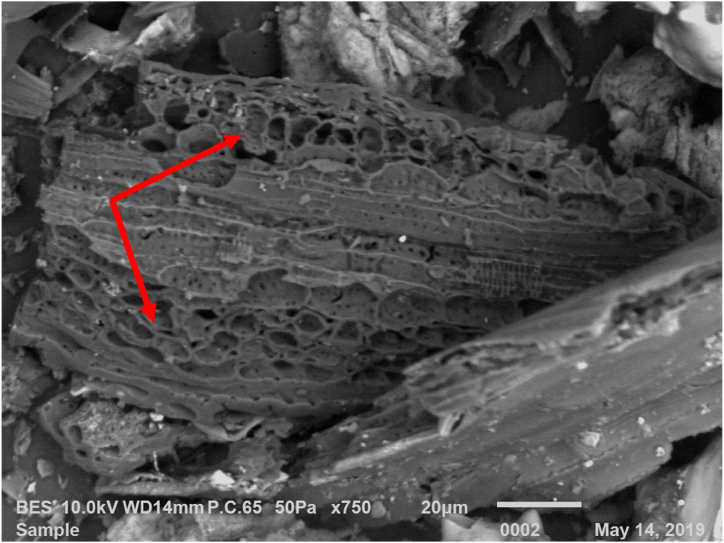
Enriched rice husk biochar surface at 700× magnification under SEM. The arrow indicates the pores of the enriched rice husk biochar.

Additionally, a SEM observation of rice husk biochar reveals that it has a bigger surface area and has a lot of pores (Fig. 1). With a greater CEC value, rice husk biochar had a pHof9.1 (Table 2). The rice husk biochar had a very high availability of P and K. Inherent cations in rice husk biochar are higher due to the increased CEC.

### 3.2 Incubation study for NH_3_ measurement

**Fig. 2:**
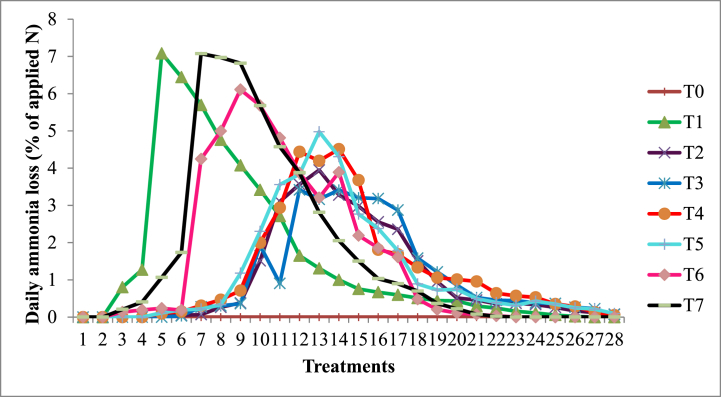
Daily ammonia volatilization over 28 days in incubation study.

The 28-day incubation study is shown in Fig. 2, which shows the significant fluctuations in the daily NH_3_ loss from urea fertilizer. The study did not include rice plants. RHB1 and RHB2 started losing NH_3_ on day 6 following the administration of urea, while RHB3 and RHB4 started losing NH_3_ on day 5. NH_3_ loss activity was not seen for CK0; on the other hand, the loss for CK1 (urea fertilizer without the addition of biochar) was observed in day 3.

Similar to CK1, NH_3_ began to volatilize in CB1 and CB2 (commercial biochar potting media) on the third day. Treatment CK1, CB1, and CB2 showed the highest NH_3_ losses on days 5, 8, and 9, respectively. Comparing CK1, CB1, and CB2 with the treatments amended with enriched rice husk biochar (RHB1, RHB2, RHB3, and RHB4), the loss in CK1, CB1, and CB2 began early and continues to cease swiftly to 1 % of the added N in the soil. The NH_3_ loss was postponed by up to 6 days in treatments RHB1 and RHB2, with the greatest losses occurring on days 13 and 12, respectively. Similarly, the loss of NH_3_ in RHB3 and RHB4 was delayed up to 5 days, with maximum loss on the 12th and 13th days. The trend of the graph shows that loss of NH_3_ peaks up and reduces gradually up to the 28th day until added urea ceases at 1 %. In comparison to urea without additives (CK1) and commercial biochar potting media (CB1 and CB2), NH_3_ loss was greatly reduced in treatments applied with enriched rice husk biochar (RHB1, RHB2, RHB3, and RHB4) (Table 3). Treatments with RHB1 and RHB2 significantly reduced NH_3_ loss by approximately 34 % compared to CK1 and RHB1. Additionally, compared to CK0, CK1, CB1, and CB2, the soil pH was significantly increased in the treatments with rice husk biochar (RHB1, RHB2, RHB3, and RHB4).

Besides, biochar had successfully chelated NH_4_^+^ and NO_3_^−^ in the soil. In comparison to treatment CK1, treatments RHB1 and RHB2 significantly retained more NH_4_^+^ in the soil (Table 3), by 73.9 % and 80.9 %, respectively, followed by treatments RHB3 and RHB4 with 49.3 % and 30.5 %, respectively. The availability of NO_3_^−^ in soil was significantly higher in treatments with enriched rice husk biochar than in CK0, CK1, CB1, and CB2.

### 3.3 Soil nutrients dynamics during rice growth in the pot experiment

Table 4 shows the selected physico-chemical characteristics of the soil under various treatments that were sampled following the harvest of the rice plant at the heading stage (70 DAT). When compared to CK0, CK1, RHB2, RHB3, and RHB4, treatment RHB1 significantly increased total N and exchangeable NH_4_^+^ in the soil (Table 4). In comparison to treatments CK0, CK1, CB1, and CB2, treatment RHB1 significantly retained more NO_3_^−^ in the soil. However, there was no significant change in the amount of NO_3_^−^ across the rice husk biochar treatments.

**Table 4:** Effects of enriched rice husk biochar on soil N, NH_4_^+^, NO_3_^−^, pH, total organic matter, total C, CEC, exchangeable acidity, exchangeable Al, and available (P, K, Ca, Mg, and Fe) at harvest (70 DAT) in pot experiment.

Soil nutrients							
Treatments	N (%)	NH_4_^+^ (ppm)	NO_3_^−^ (ppm)	pH (water)	Available P (ppm) (ds m^−1^)	Total organic matter (%)	Total C
CK0	0.07 ± 0.02^ab^	23.35 ± 2.34^a^	25.69 ± 6.18^a^	5.81 ± 0.13^a^	2.57 ± 0.68^a^	0.70 ± 0.06^a^	0.41 ± 0.03^a^
CK1	0.15 ± 0.01^c^	31.35 ± 5.24^a^	38.52 ± 2.02^a^	6.17 ± 0.03^a^	29.38 ± 3.99^b^	1.02 ± 0.19^a^	0.59 ± 0.11^a^
RHB1	0.22 ± 0.03^d^	91.07 ± 4.04^c^	84.06 ± 8.08^c^	7.02 ± 0.07^b^	119.33 ± 1.15^e^	6.30 ± 0.12^d^	3.65 ± 0.07^d^
RHB2	0.12 ± 0.06^bc^	66.55 ± 6.07^b^	63.05 ± 4.04^bc^	6.89 ± 0.09^b^	100.60 ± 1.04^d^	4.87 ± 0.09^c^	2.83 ± 0.05^c^
RHB3	0.07 ± 0.01^a^	35.03 ± 4.04^a^	46.70 ± 2.34^ab^	6.83 ± 0.06^b^	51.37 ± 0.97^c^	2.91 ± 0.59^b^	1.69 ± 0.34^b^
RHB4	0.05 ± 0.02^a^	31.52 ± 2.02^a^	2.03 ± 4.04^ab^	6.67 ± 0.07^b^	37.50 ± 3.18^b^	3.35 ± 0.27^b^	1.94 ± 0.16^b^

	**CEC**	**Exchangeable acidity (cmol kg**^**−**^**^1^)**	**Exchangeable Al** (**cmol**_**c**_**kg**^**−1**^**)**	**Exchangeable K (mg kg**^**−**^**^1^)**	**Exchangeable Ca (mg kg**^**−**^**^1^)**	**Exchangeable Mg (mg kg**^**−**^**^1^)**	**Exchangeable Fe (mg kg**^**−**^**^1^)**
CK0	2.95 ± 0.26^a^	0.33 ± 0.04^b^	0.26 ± 0.02^a^	0.36 ± 0.29^a^	0.43 ± 0.02^a^	0.08 ± 0.002^a^	0.11 ± 0.003^ab^
CK1	4.17 ± 0.27^b^	0.32 ± 0.03^b^	0.31 ± 0.03^a^	0.64 ± 0.14^ab^	0.75 ± 0.17^ab^	0.07 ± 0.001^a^	0.09 ± 0.006^a^
RHB1	9.33 ± 0.19^d^	0.17 ± 0.01^a^	0.15 ± 0.01^a^	1.44 ± 0.04^c^	4.26 ± 0.51^c^	0.04 ± 0.006^a^	0.03 ± 0.003^a^
RHB2	7.97 ± 0.09^c^	0.18 ± 0.03^a^	0.18 ± 0.11^a^	1.11 ± 0.05^bc^	1.70 ± 0.26^b^	0.05 ± 0.003^a^	0.06 ± 0.026^a^
RHB3	4.47 ± 0.26^b^	0.32 ± 0.01^b^	0.35 ± 0.02^a^	0.25 ± 0.05^a^	0.10 ± 0.01^a^	0.04 ± 0.001^a^	0.20 ± 0.026^b^
RHB4	3.80 ± 0.21^ab^	0.52 ± 0.04^c^	0.58 ± 0.09^b^	0.29 ± 0.03^a^	0.61 ± 0.06^ab^	0.05 ± 0.001^a^	0.35 ± 0.043^c^

Mean values within column with different letter(s) indicate significant difference between treatments by Tukey's test at p ≤ 0.05. Columns represent the mean values ± SE.

When compared to treatments without biochar (CK0 and CK1), the soil pH increased significantly in biochar-amended treatments (RHB1, RHB2, RHB3, and RHB4) (Table 4). The soil EC was greatly increased in treatments RHB1 and RHB2. Additionally, compared to CK0, CK1, RHB2, RHB3, and RHB4, treatment RHB1 demonstrated a significant increase in soil organic matter, total C, and CEC (Table 4).

Across treatments CK0, CK1, RHB3, and RHB4, the treatments with rice husk biochar (RHB1 and RHB2) considerably reduced the soil exchangeable acidity. In contrast to soil alone (CK0) and soil + urea (CK1), RHB1 and RHB2 did not significantly reduce the soil's exchangeable Al and Fe. Even though there was no significant reduction of Al and Fe in treatments RHB1 and RHB2, the soil available P increased significantly in comparison to the other treatments (Table 4). Similarly, RHB1 and RHB2 had increased soil exchangeable K significantly over CK0 and CK1. When compared to other treatments, treatment RHB2 (10 t ha^−1^ rice husk biochar) demonstrated a significant increase in soil exchangeable Zn, while treatment RHB1 (5 t ha^−1^) had significantly improved soil exchangeable Ca. However, there was no significant difference in exchangeable Mg retention between any of the treatments.

### 3.4 Rice husk biochar influences rice plant growth and nutrient uptake in pot experiment

Table 5 lists the rice plant's dry weight, height, number of tillers, number of panicles, and greenness. When compared to other treatments, treatment RHB1 exhibited a positive, significant increase in plant dry weight, height, tiller number, greenness, and panicle number. Similarly, RHB1 had significantly increased total N, P, K, and Mg concentrations than CK0, CK1, RHB3, and RHB4 (Table 5). Rice husk biochar added treatments (RHB1 and RHB2) did not significantly improve the concentrations of available Ca compared to CK1.

**Table 5:** Effects of enriched rice husk biochar on rice plant physical growth, total nutrient uptake, and use efficiency at harvest (70 DAT) in pot experiment.

Rice plant growth					
Treatments	**Dry weight (g)**	**Height (cm)**	**Tiller number**	**Panicle number**	**Greenness (%)**
**CK0**	7.64 ± 0.84^a^	41.94 ± 0.19^a^	2.00 ± 0.33^a^	1.00 ± 0.02^a^	100.00 ± 0.97^a^
**CK1**	22.97 ± 2.99^bc^	76.18 ± 2.92^b^	3.00 ± 0.33^a^	2.00 ± 0.33^a^	106.31 ± 3.47^a^
**RHB1**	35.22 ± 2.89^d^	92.23 ± 1.02^c^	8.00 ± 0.67^c^	7.00 ± 0.57^c^	140.32 ± 1.30^c^
**RHB2**	25.19 ± 1.97^c^	70.00 ± 0.35^b^	5.00 ± 0.58^b^	5.00 ± 0.58^b^	129.50 ± 2.15^bc^
**RHB3**	17.54 ± 1.14^bc^	73.20 ± 3.07^b^	3.00 ± 0.33^ab^	3.00 ± 0.34^a^	125.23 ± 2.84^b^
**RHB4**	14.62 ± 1.37^ab^	67.67 ± 0.98^b^	2.00 ± 0.34^a^	1.00 ± 0.33^a^	123.31 ± 3.58^b^

**Total Nutrient Uptake**					
	**N uptake (mg plant**^**−**^**^1^)**	**Total P (mg plant**^**−**^**^1^)**	**Total K (mg plant**^**−**^**^1^)**	**Total Ca (mg plant**^**−**^**^1^)**	**Total Mg (mg plant**^**−**^**^1^)**
**CK0**	2.36 ± 0.26^a^	0.003 ± 0.0001^a^	2.59 ± 1.20^a^	1.24 ± 0.80^a^	0.63 ± 2.04^ab^
**CK1**	20.35 ± 0.93^bc^	0.016 ± 0.0003^a^	51.38 ± 2.17^b^	7.12 ± 1.05^bc^	2.89 ± 1.29^b^
**RHB1**	51.42 ± 0.90^d^	0.129 ± 0.0004^c^	126.9 ± 1.26^c^	13.1 ± 1.11^d^	6.46 ± 3.60^c^
**RHB2**	26.95 ± 0.61^c^	0.076 ± 0.0007^b^	41.86 ± 2.49^b^	10.5 ± 1.18^cd^	3.12 ± 2.59^b^
**RHB3**	15.79 ± 0.35^b^	0.032 ± 0.0009^a^	32.27 ± 1.95^ab^	5.37 ± 0.70^ab^	2.18 ± 1.64^ab^
**RHB4**	12.04 ± 0.42^ab^	0.018 ± 0.0009^a^	23.47 ± 1.42^ab^	3.30 ± 0.58^ab^	1.64 ± 1.93^ab^

**Nutrient use efficiency**			
	**N use efficiency**	**P use efficiency**	**K use efficiency**
**CK0**	10.27 ± 1.93^ab^	0.007 ± 0.002^a^	27.88 ± 3.87^a^
**CK1**	28.03 ± 0.99^c^	0.072 ± 0.013^c^	71.04 ± 3.51^b^
**RHB1**	14.04 ± 0.27^b^	0.042 ± 0.003^b^	22.44 ± 3.17^a^
**RHB2**	7.68 ± 0.98^a^	0.017 ± 0.002^ab^	16.96 ± 0.87^a^
**RHB3**	5.52 ± 1.78^a^	0.009 ± 0.001^a^	11.93 ± 2.09^a^
**RHB4**	10.27 ± 1.93^ab^	0.007 ± 0.002^a^	27.88 ± 3.87^a^

Mean values within column with different letter(s) indicate significant difference between treatments by Tukey's test at p ≤ 0.05. Columns represent the mean values ± SE.

In comparison to other treatments, the enriched rice husk biochar treatment at 5 t ha^−1^ (RHB1) increased the total N, P, K, and Mg uptake by the rice plants (Table 5). The total Ca uptake by the rice plant is higher in treatments RHB1 and RHB2. Additionally, compared to other treatments, rice plant nutrient utilization efficiency was significantly increased in the rice husk biochar treatments (RHB1) (Table 5).

## 4 Discussions

### 4.1 Characteristics of enriched rice husk biochar

The two most crucial characteristics of biochar are its surface area and porosity. The high porosity level of enriched rice husk biochar is demonstrated in Fig. 1. This was closely associated with the lignin breakdown process, which was followed by an aromatic condensation reaction and a rapid release of H_2_ and CH_4_ [32,33]. Moreover, the pyrolysis process's thermal breakdown was the cause of the enriched rice husk biochar's larger surface area. The kind of biomass used to make biochar may also have an impact on the material's increased surface area. Shaaban et al. [34] state that depending on the type of feedstock used, volatile compounds are released, and the number of pores rises, increasing the surface area of biochar. Ahmad et al. [35] claim that during pyrolysis, surface area increases as a result of the breakdown of cellulose and hemicelluloses as well as the formation of channel structures. The porosity and surface area of the biochar are necessary for the soil's nutrients to be absorbed by it. Furthermore, the pH level of biochar is alkaline. The production of carbonates and the presence of inorganic alkalis are the two main causes of biochar's alkaline pH, according to Ding et al. [36]. These elements were listed by Yuan et al. [37] as the primary reasons for the alkaline pH of biochar. The alkaline pH of the biochar may also be attributed to the pyrolysis process's increase in ash content and oxygen functional group [33,38]. The kind of biomass utilized determines the CEC value, and this might lead to a high ash concentration. There is a higher proportion of ash concentration (34.4 %) in the enriched rice husk biochar employed in this investigation. This was consistent with the findings of Yang et al. [39], who reported that biochar with a higher CEC is produced from feedstock with a high ash concentration. Furthermore, the enhanced CEC may potentially be caused by the oxidation of aromatic C and the subsequent synthesis of carboxyl groups [40].

### 4.2 Ammonia volatilization incubation study

Since the urea-N fertilizer was only administered for 28 days before the study ended, rice plants were not included in the NH_3_ loss incubation investigation, and their inclusion would not have a substantial impact on the findings. Because they lack appropriate root systems, where N uptake is poor, rice plants at very young seedling stages are unable to receive the nutrients from applied urea fertilizers. It is consistent with Sun et al.'s [41] findings that, in the rice-wheat system, NH_3_ loss is typically greater during basal N fertilizer applications because of the effects of soil temperature and improper root system development in the plants. A possible explanation for this is that the crop uses more N during the active tillering vegetative stage, which is why the rate of N loss at the basal fertilization stage was highest [41]. Similar findings were made in this study, where it was found that the urea volatilizes quickly in a condition that is soil only and no biochar or rice plants. Since the rate of nutrient intake is lower at the early vegetative stage than it is at the later growth stages, it can be concluded that the NH_3_ volatilization will not be altered by the rice plants.

When compared to the other treatments in this investigation, the enriched rice husk biochar treatments RHB1 (5 t ha^−1^) and RHB2 (10 t ha^−1^) greatly reduced the NH_3_ loss (Table 3). According to Dong et al. [42], biochar's durability and gradual breakdown process allow it to improve NH_4_^+^ adsorption and decrease NH_3_ volatilization even after three years. This demonstrates how the porosity, stability, and recalcitrance of biochar facilitate ion adsorption. Because of its larger surface area and pores, the rice husk biochar utilized in this study is better at adsorbing NH_4_^+^ and NO_3_^−^ ions, which reduces the release of NH_3_.

Additionally, it was supported by research results by Chen et al. [32], which demonstrated that the porosity and greater surface area of biochar accelerated NH_4_^+^ adsorption over NH_3_ volatilization. Due to the adsorptive ability of the biochar, even the soil pH rise in the rice husk biochar treatments (Table 3) does not cause NH_3_ volatilization. According to a prior study, an increase in soil pH (>8.5) accelerates the volatilization of NH_3_ because of the ammonification reaction, in which OH-binds with NH_4_^+^ to generate NH_3_ [43]. In this work, the addition of rice husk biochar results in a pH that is nearly neutral, and a small rise has no effect on the volatilization of NH_3_. The fact that biochar adsorbs NH_4_^+^ ions onto their exchange sites prior to their reaction with OH^−^ may be the cause of this. Furthermore, the H^+^ ions of acid functional groups on the surface of the biochar may protonate NH_3_ to generate NH_4_^+^ because of the higher CEC (>66.66 cmolc kg^−1^) of rice husk biochar [44,45]. The results of the study, which indicated that treatments applied with enriched rice husk biochar substantially maintained more NH_4_^+^ and NO_3_^−^ ions in the soil than those that used commercial biochar potting medium (CB1 and CB2) and treatments without biochar (CK0 and CK1) (Table 3).

The ability of rice husk biochar to decrease NH_3_ loss is greater than that of commercial biochar potting media. This may be because the biochar is physically enhanced with chicken manure slurry, which boosts its nutrient content and adsorption capacity. Because biochar decomposes more slowly than other materials, the nutrients it has absorbed tend to release gradually over time. The overwhelming weight of data points to a 3.5-year slow rate of biochar decomposition in soil [46]. Due to the fact that biochar releases adsorbed nutrients gradually over time, it improves soil nutrient levels and facilitates plant absorption.

### 4.3 Soil nutrients improvement

The experiment's findings demonstrated a correlation between the enhanced biochar and the soil's N, NH_4_^+^, and NO_3_^−^ concentrations. When compared to other treatments, the soil total N, NH_4_^+^, and NO_3_^−^ in treatments treated with 5 t ha^−1^ enriched rice husk biochar increased significantly. This rise may be attributed to the porosity of the biochar. The physical entrapments in the biochar's pores provide the enriched rice husk biochar a great capacity to sorb ions. Additionally, biochar's greater surface area helps the soil's ability to absorb total N, NH_4_^+^, and NO_3_^−^ (Fig. 1). Because of its porosity and higher surface area, biochar generated at a lower temperature (<500 °C) has the greatest capacity to promote NH_4_^+^, and NO_3_^−^ formation and adsorption [47,48]. The adsorption of N, NH_4_^+^, and NO_3_^−^ was also found to be enhanced by the biochar's micropores and high surface area charge, according to Mavi et al. [49] and Guerena et al. [50]. The increased CEC of rice husk biochar (66.6 cmolc kg^−1^) was similarly linked to the efficient ion retention.

The soil pHhadimprovedsubstantiallyoverCK1 following the application of enhanced rice husk biochar (Table 4). With an ash level of 34.4 %, the enriched rice husk biochar employed in this study proved useful in enhancing acidic soil. The pH of the soil had increased because of the ash concentration. The exchange of protons (H^+^) between the soil and enriched rice husk biochar may also be linked to the elevated pH of the soil. A sequence of proton consumption processes caused by the application of enhanced rice husk biochar neutralized the acidic soil. The results of this investigation are consistent with those of Ch'ng et al. [51], which found that the addition of organic amendments raised the pH of the soil through the process of proton exchange. The enriched rice husk biochar's natural base cations were likewise linked to the soil pH increase. As the biochar breaks down, base cations including Na, Ca, Mg, and K are released into the soil. This solubilization process consumes protons in the soil and lowers the acidity of the soil.

Furthermore, it was observed that the addition of enhanced rice husk biochar to the treatment resulted in a considerable reduction of soil exchangeable acidity. The higher pH of the soil is partially responsible for the finding. Lower soil Al and Fe levels are associated with decreased exchangeable acidity and pH levels. Because insoluble Al and Fe hydroxides occur in soil with a higher pH, the soil Al and Fe will be lower in that soil [52]. In contrast, although there is no discernible decrease in soil Al and Fe, there is no interference of these elements in P fixation or soil pH in this investigation. With rice husk biochar, treatments RHB1 and RHB2 had noticeably more accessible soil P. This showed that the addition of enriched rice husk biochar renders the activity of P fixation by Al and Fe. The adsorption of ${\text{PO}}_{4}^{3}$^-^ ions onto the enriched rice husk biochar may be the cause of the rise in soil P. Biochar's polar and non-polar surface sites help ions like NH_4_^+^, and NO_3_^−^, and ${\text{PO}}_{4}^{3}$^-^ adsorb onto its exchange sites [12]. This also lined up with a study that was carried out by Sarkhot et al. [53]. The gradual release of ${\text{PO}}_{3}^{-}$ ions that were absorbed from chicken manure slurry throughout the enrichment process may also have had a role in the increase in soil P.

Enhanced rice husk biochar application resulted in a considerable increase in soil organic matter and total C content. This may be because the biochar contains aromatic compounds that make it more stable in soil and prevent microbial deterioration [54,55]. An increase in soil total C is correlated with an increase in soil organic matter. Biochar is a C-rich, recalcitrant substrate that resists breakdown, increasing the total C content of the soil in the process [56,57]. Soil EC increases when enriched rice husk biochar is added to treatments. This resulted from the rice husk biochar's natural higher-soluble salt content. The breakdown of cations from the surface of the biochar may also be the cause of the increase in the EC of the soil. When enriched rice husk biochar was applied to the soil, the CEC of the soil increased. This could be connected to the enriched rice husk biochar's high surface area and porosity. The increase in soil CEC is also linked to the slow oxidation of biochar, which oxygenates the surface functional group and boosts cation sorption from the soil [59,60]. Furthermore, the exchangeable K, Ca, and Zn were raised by the addition of enriched rice husk biochar. The cation increase is linked to both the soil CEC and the higher ash concentration in the rice husk biochar, which facilitates the release of Ca, Zn, and K [53,58]. The pH of the soil increased after alkaline rice husk biochar was applied, and this was correlated with a rise in soil CEC. The soil CEC rises as a result of an increase in soil pH because soil colloids' negative charge facilitates cation binding.

### 4.4 Rice plant growth performance, nutrient uptake, and nutrient use efficiency

The rice plants treated with rice husk biochar showed a considerable increase in height, number of tillers, panicles, and degree of greenness. Improved soil chemical characteristics lead to a reduction in soil acidity and an increase in plant nutrient availability (N, P, and K), which improves rice plant growth performance. In treatments using rice husk biochar, the dry weight of the rice plants rose noticeably.

The efficiency with which the rice plants absorbed and utilized nutrients was greatly enhanced by the addition of rice husk biochar. Zhang et al. [61] and Shen et al. [62] reported that the application of biochar greatly increased the plant's N consumption efficiency. Because biochar has a significant ability to acquire and store N over an extended length of time, the agricultural biomass helped to boost the efficiency of N usage [61]. The N, P, K, Ca, and Mg were much more readily absorbed by the rice plant in RHB1 (5 t ha^−1^ enhanced rice husk biochar). This finding corroborates Table 5, which indicates that the best utilization efficiency was found in RHB1, N, P, and K compared to other treatments.

The two main soil nutrients that plant roots absorb are NH_4_^+^, and NO_3_^−^, with NH_4_^+^ being preferred by rice. Due to the rice husk biochar's ability to adsorb NH_4_^+^ ions and progressively release them for rice plant N uptake, the addition of biochar in this study positively regulates the nutrients and increases N use efficiency. NH_4_^+^, and NO_3_^−^ production and adsorption over NH_3_ are increased by rice husk biochar. A higher rate of plant N uptake and usage efficiency was linked to the ability of organic amendments to reduce NH_3_ volatilization [1]. Moreover, to meet the requirements of the various stages of rice plant growth, the adsorbed N, NH_4_^+^, and NO_3_^−^ at biochar exchange sites were progressively released into the soil.

The P, K, Ca, and Mg concentrations in rice plants, as well as their absorption and usage efficiency, were all significantly higher in RHB1 compared to the other treatments. The addition of enhanced rice husk biochar effectively increased the adsorption of PO_4_^3−^ onto its exchange sites by reducing fixation by Al and Fe. The PO_4_^3−^ ions will not be released by the biochar right away, which will help the plant roots absorb P. The rice plant eventually developed longer roots, which increased the intake of P, K, Ca, and Mg. The enrichment of rice husk biochar with nutrient-rich chicken manure slurry is also partially responsible for the increase in N, P, K, Ca, and Mg absorption in rice plants. Because rice husk biochar has a complex and stable structure, it breaks down gradually, releasing the nutrients it has absorbed from chicken manure slurry into the soil for plant uptake.

## 5 Conclusion

In order to promote plant development, the current study demonstrates the noteworthy influence of biochar amendments on soil quality indicators. By controlling fertilizer usage, the addition of enriched rice husk biochar to the soil has the potential to boost soil nutrients and rice plant growth, hence enhancing agricultural sustainability. Enriched rice husk biochar greatly reduced NH_3_ loss at a 5 t ha^−1^ application rate by retaining more NH_4_^+^, and NO_3_^−^ ions in the soil, which promotes effective plant N uptake and utilization. The pH of the soil is raised and its levels of macro- and micronutrients are enhanced when rice plants are cultivated in soil that has been supplemented with rice husk biochar. These two factors directly support the physical growth of rice plants. The application of 5 t ha^−1^ of enriched rice husk biochar considerably enhanced the rice plant's capacity to produce more dry matter, absorb nutrients, and utilize those nutrients more efficiently. To sum up, applying enriched rice husk biochar has the potential to greatly lower N loss and retain more nutrients in the soil for uptake by rice plants. To reduce NH_3_ loss from applied urea fertilizer, rice cultivation agronomic practices could include the use of rice husk biochar at a rate of 5 t ha^−1^; however, a long-term field experiment is required to validate the results. The effectiveness of the enriched rice husk biochar in reducing NH_3_ loss from urea fertilizer, boosting soil nutrients, and promoting rice plant development is now being evaluated and validated by field tests.

## CRediT authorship contribution statement

**Gunavathy Selvarajh:** Writing – original draft, Software, Resources, Project administration, Methodology, Investigation. **Huck Ywih Ch'ng:** Writing – original draft, Validation, Supervision, Software, Resources, Project administration, Conceptualization. **Norhafizah Md Zain:** Writing – original draft. **Lee Seong Wei:** Writing – review & editing. **Jeng Young Liew:** Software. **Siti Nuurul Huda Mohammad Azmin:** Visualization, Validation, Supervision. **Laila Naher:** Writing – original draft. **Palsan Sannasi Abdullah:** Writing – review & editing. **Osumanu Haruna Ahmed:** Writing – review & editing. **Mohamadu Boyie Jalloh:** Writing – review & editing. **Issariyaporn Damrongrak:** Writing – review & editing.

## Declaration of competing interest

The authors declare the following financial interests/personal relationships which may be considered as potential competing interests:Ch'ng huck wyih reports financial support was provided by Malaysia 10.13039/501100002385Ministry of Higher Education. Lee Seong Wei is an Heliyon editor.
